# Monitoring Compliance With Local Policy: A Re-Audit of Senior Psychiatrist Review of New Admissions Within 48 Hours at Mental Health Unit, Forth Valley Royal Hospital

**DOI:** 10.1192/bjo.2026.11656

**Published:** 2026-06-30

**Authors:** Hannah Driver, Ashwin Moothedath Asok

**Affiliations:** NHS Forth Valley, Falkirk, United Kingdom

## Abstract

**Aims:**

As per NHS Forth Valley Psychiatry Emergency plan 2023, any patient admitted to the mental health unit should have a senior review within 48 hours of admission, regardless of the day of the week. A senior review is defined as either a higher trainee, i.e. ST4-6, or a consultant psychiatrist. Previous audits have reviewed compliance of this in the years 2021 and 2022. The aim of this re-audit was to review the compliance of the 48-hour senior review policy between 2023 to 2025.

**Methods:**

Electronic patient records of patients admitted to five mental health wards in Forth Valley Royal Hospital were reviewed to determine whether there was a clear documentation of senior review within 48 hours of admission, in line with the existing policy. This was done biannually for the duration between August 2022-- August 2025 in a retrospective manner.

**Results:**

In 2025, all wards achieved 100 percent compliance with 48 hour senior review policy. Overall average of compliance in duration from Aug 2022 - February 2023 was 97 percentage which increased to 100 percentage between August 2024 - August 2025. One ward which showed low compliance (78%) in the time period August 2022 - February 2023, demonstrated progressive improvement in the re-audit cycles in February 2024 (89%), August 2024 (90%) and further 100 % compliance in both audit cycles in 2025. Interestingly, the Intensive Psychiatric Care Unit, which has the most acutely unwell patients, had showed full compliance with the 48-hour policy in all audit cycles since 2021.

**Conclusion:**

Sustained improvement in compliance to 48 hours senior review policy was demonstrated by this audit, with all wards achieving full compliance in2025. To improve quality of care, a new audit process is being planned for the introduction of a standardised senior review template. Additionally, discussions are ongoing within the trust to adopt a 24-hour senior review policy which is the standard practice in medical wards.

